# Venous thromboembolism in cancer surgery: A report from the nationwide readmissions database^☆^^☆☆^

**DOI:** 10.1016/j.sopen.2022.04.005

**Published:** 2022-05-07

**Authors:** Chelsea S. Pan, Yas Sanaiha, Joseph Hadaya, Cory Lee, Zachary Tran, Peyman Benharash

**Affiliations:** Cardiovascular Outcomes Research Laboratories, Division of Cardiac Surgery, David Geffen School of Medicine at UCLA, Los Angeles, CA

## Abstract

**Background:**

The present study characterized the incidence of venous thromboembolism in a contemporary cohort of surgical oncology patients and its association with index hospitalization and postdischarge outcomes.

**Methods:**

Adults undergoing 7 major thoracic and abdominal cancer resections were identified in the 2016–2019 Nationwide Readmissions Database. Multivariable models stratified by operative subtype were developed to evaluate the association of venous thromboembolism with outcomes of interest.

**Results:**

Of an estimated 436,368 patients, venous thromboembolism was identified in 9,811 (2.2%) patients during index hospitalization. Esophageal (4.1%) and gastric (4.1%) resections exhibited the highest rates of venous thromboembolism, whereas pulmonary resection (1.0%) the lowest. Following adjustment, cancer resection type demonstrated the strongest association with venous thromboembolism development among all factors analyzed (adjusted odds ratio: 3.13, 95% confidence interval: 2.60–3.78). Diagnosis of venous thromboembolism was associated with increased mortality (10.2%, 95% confidence interval: 9.4–11.1 vs 1.7, 95% confidence interval: 1.6–1.7) and prolonged index hospital stay (19.5 days, 95% confidence interval: 19.1–20.0 vs 7.5, 95% confidence interval: 7.4–7.5). Of patients who survived index hospitalization, venous thromboembolism occurrence was associated with increased risk of nonhome discharge (56.4%, 95% confidence interval: 54.7–58.0 vs 14.4, 95% confidence interval: 14.2–14.7) and readmission (30.0%, 95% confidence interval: 28.5–31.1 vs 16.9, 95% confidence interval: 16.7–17.1). Additionally, venous thromboembolism substantially increased index hospitalization ($40,000, 95% confidence interval: $38,000–$42,000) and readmission costs ($3,200, 95% confidence interval: $1,700–$4,700).

**Conclusion:**

Rates of venous thromboembolism remain high in surgical oncology patients, with cancer resection type as a major predictor of venous thromboembolism incidence. Venous thromboembolism was associated with inferior clinical and financial outcomes that extended beyond discharge. These findings underscore the importance of continued vigilance and procedure-specific prophylaxis measures.

## INTRODUCTION

The landscape of venous thromboembolism (VTE) in cancer patients has evolved significantly over the past 2 decades. Despite advances in surgical technique, perioperative management, and prophylaxis measures, the incidence of VTE among hospitalized cancer patients has increased [1]. In fact, VTE is considered the leading cause of mortality among those receiving operations for cancer with a reported incidence of up to 50% [2,3]. The risk of postoperative VTE in cancer patients may be particularly increased because of exacerbation of immobility in addition to hypercoagulability and endothelial injury [2,4,5]. Improved diagnostics, exposure to neoadjuvant therapies, and increased complexity of cancer operations have also been cited as reasons for the observed phenomenon in recent years [4,5].

Although much of the literature on VTE during hospitalization for cancer surgery is dated, recent work by Mallick et al and Jarvis et al has demonstrated that the risk of VTE extends beyond the immediate postoperative period [6,7]. Accordingly, recommendations from several organizations including the American Society of Clinical Oncology and the American Society of Hematology have specifically targeted VTE prophylaxis among cancer patients in both the inpatient setting and postdischarge [8,9,10]. However, it is now known that several factors including the type of cancer and operative approach influence the risk of VTE [11]. Moreover, greater adoption of minimally invasive surgery has led to earlier postoperative ambulation, reduced narcotic use, and less immobilization, which may influence VTE rates [[12], [13], [14], [15], [16]]. Recent changes in practice patterns, including early initiation of prophylaxis as well as laboratory monitoring of anti-factor Xa, may also impact VTE rates and associated outcomes [17]. Given these notable changes to the care of surgical oncology patients, updated studies are needed to evaluate the impact of VTE in this population.

The present study sought to characterize the incidence of VTE among operative hospitalizations to treat abdominal and thoracic malignancies in a national and contemporary cohort. We hypothesized VTE to be associated with inferior clinical and financial outcomes during index hospitalization and readmission up to 90 days after discharge.

## METHODS

### Study Design and Data Source

The present study was a retrospective cross-sectional study of the 2016–2019 Nationwide Readmissions Database (NRD), the largest nationally representative, all-payer readmissions database in the United States. Maintained as part of the Healthcare Utilization Project (HCUP), the NRD accrues data from 28 participating states and provides accurate estimates for approximately 58% of all US hospitalizations [18]. Patients are followed across hospitalizations using a unique patient identifier, allowing for tracking of readmissions within each calendar year. This study was deemed exempt from full review by the Institutional Review Board at the University of California, Los Angeles.

### Patient Selection

We identified all adult elective hospitalizations for major thoracic and abdominal cancer operations: esophageal, pulmonary, gastric, pancreatic, hepatobiliary, colonic, and rectal resections with a corresponding cancer diagnosis. Previously published *International Classification of Disease, Tenth Revision* (*ICD-10*) Procedure Codes were used to identify selected operations, and *ICD-10* Diagnosis Codes were used to corroborate corresponding cancer diagnoses [6]. We included discharges up to October of each calendar year to allow for adequate 90-day follow-up for all patients in the study. Patients undergoing more than 1 operation as well as those with a history of chronic VTE diagnoses were excluded from our cohort. Additionally, records with missing key demographic or outcome data were excluded (Supplementary Fig 1).

### Variable Definitions and Study Outcomes

The diagnoses of acute pulmonary embolism and deep venous thromboembolism, collectively venous thromboembolism (VTE), were ascertained using previously published *ICD-10* codes [6]. Patient and hospital characteristics were defined according to the NRD Data Dictionary and included age, sex, and primary payer along with hospital location and teaching status [18]. Comorbidities were defined using *ICD-10* codes and the Elixhauser Comorbidity Index, a composite score of 30 common conditions used to quantify the burden of chronic disease [19]. We excluded comorbidities of metastatic cancer and solid tumor from the Elixhauser Index because all patients had a cancer diagnosis. Operative type was designated as open or minimally invasive. Hospitals were classified into low, medium, and high volume based on tertiles of annual combined volume of cancer operations of interest. Nonhome discharge included transfer to skilled nursing facilities, rehabilitation facilities, and other acute care centers. Hospitalization charges were calculated from cost-to-charge ratios provided by HCUP and adjusted for inflation using the 2019 Personal Consumption Expenditure Health Price Index [20]. Readmissions to NRD participating hospitals were tracked within 1 calendar year and censored at 90 days.

The primary outcome of interest was the association of VTE with index hospitalization mortality stratified by cancer resection type and related hospital length of stay (LOS) and costs. Secondary outcomes included the influence of VTE on postdischarge outcomes, comprising nonhome discharge, nonelective readmissions, as well as readmission-associated LOS and costs.

### Statistical Analysis

All statistical analyses were performed with Stata 16.0 (StataCorp, LLC, College Station, TX) using HCUP survey-specific methods to account for clustering. Continuous variables are reported as medians with interquartile range (IQR) and categorical variables as frequencies. Mann–Whitney *U* and Pearson *χ*^2^ tests were used to compare continuous and categorical variables, respectively. Multivariable logistic and linear models were developed to evaluate VTE incidence and its association with selected outcomes. The least absolute shrinkage and selection operator, a regression algorithm that reduces overfitting and improves out-of-sample reliability, guided variable selection [21]. The final model was optimized using receiver operating characteristics when appropriate. Outputs of logistic model are reported as adjusted odds ratios (AORs) and risk-adjusted values were calculated using the Stata *margins* command and are reported as estimates with 95% confidence intervals (95% CIs). The Nelson–Aalen method was used to estimate the cumulative hazard of readmission within 90 days of discharge [22].

## RESULTS

### Patient Characteristics

Of an estimated 436,368 patients meeting inclusion criteria, 9,811 (2.2%) had a concomitant diagnosis of VTE during index hospitalization. Compared to others, the VTE cohort was older (71 years [62–79] vs 68 [60–76], *P* < .001) and had a higher Elixhauser Comorbidity Index (3 [4–6] vs 2 [1–4], *P* < .001, Table 1). More than 90% of oncologic resections studied were performed at metropolitan hospitals, with a higher proportion of VTE patients receiving care at teaching institutions (79.2% vs 77.5, *P* < .001). As expected, patients with VTE more commonly underwent open (76.2% vs 55.9, *P* < .001) rather than minimally invasive procedures. Esophageal (4.1%) and gastric (4.1%) resections exhibited the highest rates of VTE, whereas pulmonary resection (1.0%) had the lowest (Table 2).

**Table 1:** Patient and hospital characteristics

	*nVTE* *(*n *= 426,557)*	*VTE* *(*n *= 9,811)*	P *value*
Median age [IQR]	68 [60–76]	71 [62–79]	<.001
Female sex, %	49.6	46.2	<.001
Median Elixhauser Comorbidity Index [IQR]	2 [1–4]	3 [4–6]	<.001
Income, %			.57
0–25th quartile	25.3	26.0	
26th–50th quartile	27.3	26.6	
51st–75th quartile	25.5	25.8	
76th–100th quartile	21.9	21.6	
Payer, %			<.001
Private	29.1	22.2	
Medicare	60.4	65.8	
Medicaid	7.0	7.8	
Self-pay or uninsured	3.5	4.1	
Operation type, %			<.001
Open	55.9	76.2	
Minimally invasive	44.1	23.8	
Resection type, %			<.001
Esophageal	1.9	3.6	
Pulmonary	28.5	13.1	
Gastric	4.6	8.5	
Pancreatic	6.5	9.5	
Colonic	48.5	55.9	
Rectal	6.8	5.4	
Hepatobiliary	3.1	4.0	
Bed size, %			.02
Large	63.4	61.9	
Medium	23.8	25.7	
Small	12.8	12.4	
Location and teaching status, %			<.001
Nonmetropolitan	5.9	3.7	
Metropolitan nonteaching	16.6	17.1	
Metropolitan teaching	77.5	79.2	
Hospital volume, %			.05
High	31.2	32.9	
Medium	31.2	29.9	
Low	37.6	37.2	

*nVTE*, no venous thromboembolism.

**Table 2:** VTE incidence by cancer resection

	*No. of patients*	*DVT (%)*	*PE (%)*	*VTE (%)*
Esophageal	8,502	264 (3.1)	126 (1.5)	349 (4.1)
Pulmonary	123,044	834 (0.7)	598 (0.5)	1,288 (1.0)
Gastric	20,417	601 (2.9)	301 (1.5)	829 (4.1)
Pancreatic	28,654	728 (2.5)	276 (1.0)	933 (3.3)
Colonic	212,560	3,893 (1.8)	2,511 (1.2)	5,489 (2.6)
Rectal	29,681	403 (1.4)	168 (5.7)	532 (1.8)
Hepatobiliary	13,511	296 (2.2)	159 (1.2)	392 (2.9)
Overall cohort	436,368	7,020 (1.6)	4,139 (0.95)	9,811 (2.2)

*DVT*, deep venous thromboembolism; *PE*, pulmonary embolism

### Factors Associated With VTE

**Fig 1:**
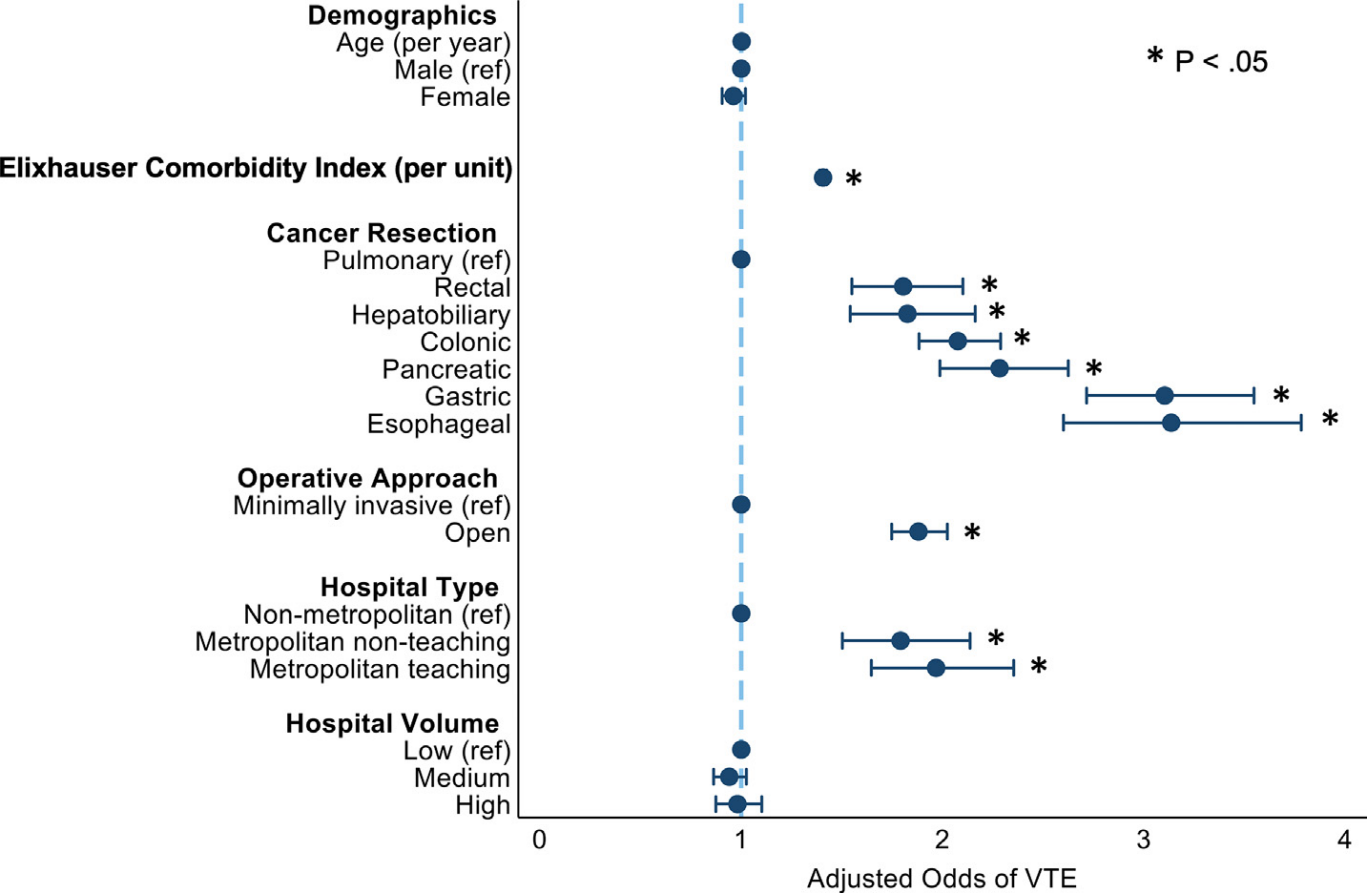
Factors independently associated with VTE development. *Ref*, reference.

Following risk adjustment, cancer resection type demonstrated the strongest association with development of VTE among all covariates analyzed, as shown in Figure 1. With pulmonary resections as reference, all other operations exhibited a 2–3-fold increase in the odds of VTE. Esophageal (AOR: 3.14, 95% CI 2.60–3.78) and gastric resections (AOR: 3.10, 95% CI 2.71–3.55) had the highest odds of VTE among all operations considered. Other factors associated with increased odds of VTE included greater Elixhauser Comorbidity Index (AOR: 1.41/unit, 95% CI: 1.39–1.42), open operative approach (AOR: 1.88, 95% CI: 1.75–2.02, ref.: minimally invasive), and care received at metropolitan teaching hospitals (AOR: 1.97, 95% CI: 1.65–2.35, ref.: nonmetropolitan).

### Outcomes at Index Hospitalization

**Fig 2:**
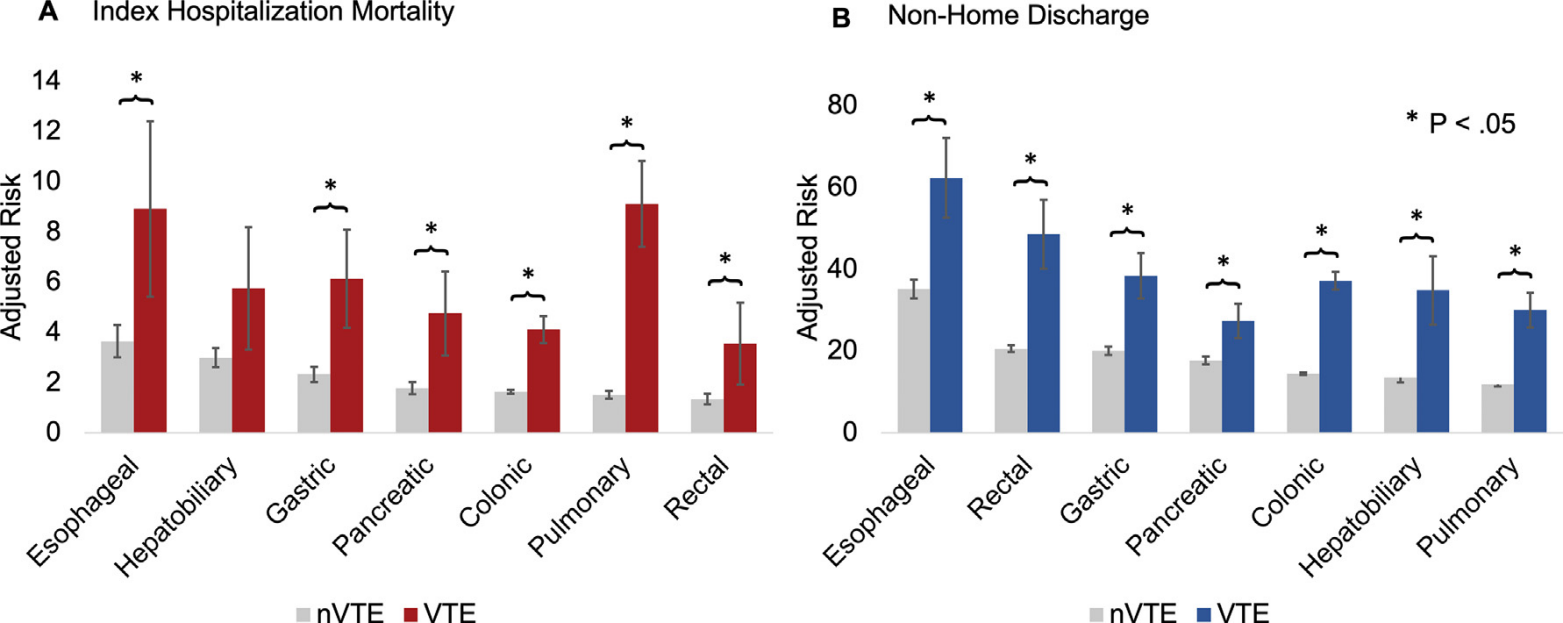
Adjusted risk of index hospitalization mortality (A) and nonhome discharge (B) by resection type. *nVTE*, no venous thromboembolism.

Unadjusted comparisons of outcomes stratified by VTE occurrence are shown in Supplementary Table 1, Supplementary Table 2. Following risk adjustment, diagnosis of VTE was associated with increased probability of mortality across all operative groups with the exception of hepatobiliary procedures, in which the difference did not reach statistical significance (Fig 2). As shown in Figure 2, esophageal resection had the highest baseline mortality (3.7%, 95% CI: 3.0–4.3), which was further exacerbated by the development of VTE (8.9%, 95% CI: 5.4–12.4). Although pulmonary resections had a low baseline mortality (1.5%, 95% CI: 1.4–1.7), occurrence of VTE increased this risk dramatically to 9.1% (95% CI: 7.4–10.8).

Development of VTE increased duration of index hospitalization by 12.0 days (7.5 days, 95% CI: 7.4–7.5 to 19.5, 95% CI: 19.1–20.0) when averaging across all operations. Stratification by operative subtype demonstrated the largest incremental increase in LOS for proctectomy (7.9 days, 95% CI: 7.7–8.0 to 20.7, 95% CI: 18.3–23.1) and esophagectomy (13.0 days, 95% CI: 12.6–13.4 to 24.8, 95% CI: 21.7–27.8), as shown in Table 3. Presence of VTE as a diagnosis was associated with a $40,000 ($28,600, 95% CI: $28,200–$29,000 to $68,400, 95% CI: $66,100–$70,700) increase in index hospitalization costs when averaging across all operations.

**Table 3:** Risk adjusted hospital LOS and cost stratified by VTE incidence for index hospitalization and readmission

	*Index LOS (d)* *[95% CI]*		*Index cost ($1,000)* *[95% CI]*		*Readmission LOS (d)* *[95% CI]*		*Readmission cost ($1,000)* *[95% CI]*	
	*nVTE* *(*n *= 426,557)*	*VTE* *(*n *= 9,811)*	*nVTE* *(*n *= 426,557)*	*VTE* *(*n *= 9,811)*	*nVTE* *(*n *= 70,829)*	*VTE* *(*n *= 2,634)*	*nVTE* *(*n *= 70,829)*	*VTE* *(n = 2,634)*
Esophageal	13.0 [12.6, 13.4]	24.8 [21.7, 27.8]	55.9 [53.9, 58.0]	111.2 [95.8, 126.6]	8.3 [7.5, 9.2]	8.0 [6.1, 9.9]	23.9 [20.8, 27.1]	18.4 [13.1, 23.8]
Pulmonary	6.5 [6.5, 6.6]	16.7 [15.3, 18.1]	28.3 [27.8, 28.7]	70.2 [64.0, 76.4]	5.9 [5.8, 6.1]	8.5 [6.6, 10.4]	14.7 [14.2, 15.3]	26.7 [18.5, 34.9]
Gastric	10.5 [10.3, 10.7]	20.0 [18.6, 21.5]	39.4 [38.3, 40.5]	78.6 [70.3, 87.0]	8.2 [7.7, 8.6]	8.0 [6.7, 9.4]	21.5 [19.4, 23.5]	23.7 [16.8, 30.7]
Pancreatic	9.5 [9.3, 9.6]	17.2 [15.5, 18.8]	39.8 [38.7, 40.8]	70.3 [63.3, 77.4]	6.8 [6.5, 7.0]	7.2 [5.8, 8.6]	17.3 [16.3, 18.2]	18.3 [14.6, 21.9]
Colonic	7.3 [7.3, 7.4]	15.9 [15.4, 16.4]	24.8 [24.4, 25.2]	50.2 [48.1, 52.4]	6.5 [6.4, 6.6]	6.9 [6.3, 7.5]	15.2 [14.9, 15.6]	15.5 [14.0, 16.9]
Rectal	7.9 [7.7, 8.0]	20.7 [18.3, 23.1]	29.3 [28.7, 30.0]	72.5 [62.6, 82.4]	6.8 [6.5, 7.1]	8.4 [6.5, 10.4]	15.2 [14.1, 16.3]	17.5 [13.0, 22.0]
Hepatobiliary	7.3 [7.1, 7.5]	18.8 [16.5, 21.2]	37.2 [35.7, 38.6]	87.7 [74.7, 100.7]	6.1 [5.7, 6.5]	6.6 [4.9, 8.3]	17.1 [15.7, 18.5]	17.9 [13.0, 22.8]
Overall cohort	7.5 [7.4, 7.5]	19.5 [19.1, 20.0]	28.6 [28.2, 29.0]	68.4 [66.1, 70.7]	6.5 [6.4, 6.6]	7.8 [7.3, 8.3]	16.0 [15.6, 16.3]	19.2 [17.6, 20.7]

### Postdischarge Outcomes

**Fig 3:**
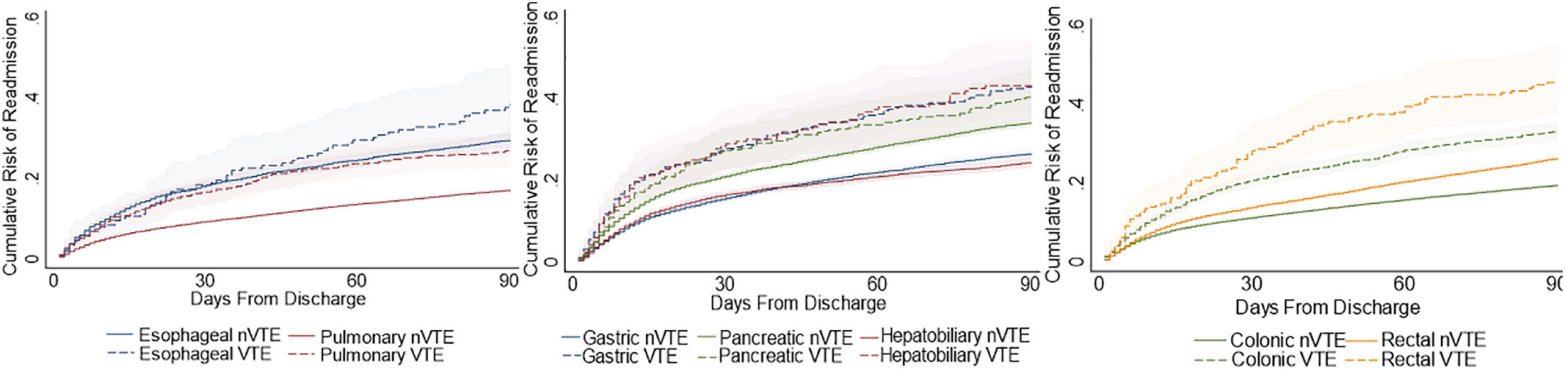
Cumulative risk of readmission within 90 days of discharge by resection type. Log-rank *P* < .05 for all operations except esophageal.

Among patients surviving index hospitalization, development of VTE was associated with overall increased risk of discharge to a rehabilitation or intermediate facility (56.4%, 95% CI: 54.7–58.0 vs 14.4, 95% CI: 14.2–14.7). This association was most profound following esophagectomy, in which VTE increased the probability of nonhome discharge by 27.2% (35.2%, 95% CI: 32.9–37.5 vs 62.4, 95% CI: 52.6–72.1, Fig 2). Additionally, VTE was linked to increased readmission within 90 days of discharge when considering all operations (30.0%, 95% CI: 28.5–31.1 vs 16.9, 95% CI: 16.7–17.1). Cumulative risk of readmission over time stratified by the presence of VTE incidence is shown in Figure 3. Nelson–Aelan analysis demonstrated all operative subgroups experienced higher cumulative risk of readmission following the occurrence of VTE, except for esophagectomy where statistical significance was not reached.

The VTE cohort was not only more likely to be readmitted but also faced increased risk of mortality at rehospitalization (5.5%, 95% CI: 4.4–6.7 vs 3.7, 95% CI: 3.5–4.0). Further analysis revealed such disparity to be primarily driven by higher mortality among patients who underwent pulmonary resection and developed VTE (10.5%, 95% CI: 6.1–15.0 vs 4.0, 95% CI: 3.5–4.4). Finally, diagnosis of VTE on index hospitalization was associated with a 1.3-day increase in readmission LOS (6.5 days, 95% CI: 6.4–6.6 to 7.8, 95% CI: 7.3–8.3) and $3,200 ($16,000, 95% CI: $15,600–$16,300 to $19,200, 95% CI: $17,600–$20,700) increase in hospitalization costs across all operations.

## DISCUSSION

In this large-scale and contemporary study, we found VTE to remain a relevant and serious complication following major cancer operations. By utilizing the NRD, we were able to assess VTE-associated outcomes during both index hospitalization and readmission. Among hospitalizations for oncologic resection of 7 common thoracic and abdominal malignancies, we noted an overall VTE incidence of 2.2%. The odds of developing VTE varied greatly among operation types and were highest in esophageal and gastric resections. We found that VTE was associated with increased mortality and hospital length of stay across all admissions. Importantly, development of VTE was linked with markers of increased resource use including hospitalization costs, nonhome discharge, and readmissions. Given the ongoing prevalence and impact of perioperative VTE, several of our findings warrant further discussion.

Both cancer and surgery are now recognized as independent risk factors for VTE [2,4,5]. A combination of immobility, release of tissue factor, and reduced fibrinolysis is thought to contribute to this increased risk [5]. Although a body of previous literature has characterized the risk of VTE among surgical cancer patients, contemporary data are generally lacking [1,2,11]. This is particularly relevant because a number of parallel advances in cancer care and surgical technology have occurred in recent years [23]. Patients are now more likely to receive neoadjuvant therapies, and more aggressive selection criteria have resulted in operative management of more advanced disease [[24], [25], [26], [27]]. Much focus has been paid to reducing the trauma of surgery and enhancing recovery pathways which, in turn, may reduce the risk of VTE [28,29]. Importantly, societal guidelines have recommended pharmacologic prophylaxis against VTE in all surgical patients with cancer [8,9,10]. In this context, we found 2.2% of the cohort in this study to develop VTE during hospitalization with an incident mortality of 10.2% across all operative groups. In comparison, a prior national study of cancer patients by Trinh et al over a decade ago noted an overall VTE rate of 1.3% and mortality of 12% [1]. The paradoxical increase in risk of VTE noted in our study may represent, on one hand, changes in patient and operative complexity and, on the other, increased sensitivity to diagnosis. Although we were unable to assess adherence to VTE prophylaxis or enhanced recovery protocols in the present work, such factors should be optimized and reexamined in future work.

An important finding of the present work is the variability in the incidence of VTE and its influence on outcomes of interest across various operation types. Previously, De Martino et al found rates of VTE ranging from 0.3% to 7.3% among abdominal oncologic operations, with the highest incidence following esophagostomy, cystectomy, and pancreatectomy [11]. Although the present analysis found similarly high rates of VTE among patients undergoing esophagectomy and pancreatectomy, we also noted a high risk of VTE among gastrectomy patients with lobectomy patients as reference. Notably, when we examined patient, operative, and hospital factors independently associated with the development of VTE, we found cancer resection type to afford the greatest influence. Although resection type is dictated by the tumor location and biologic characteristics, our data suggest a reduction in the risk of VTE with minimally invasive operations, which may facilitate early mobility and reduce tissue trauma. Expectedly, development of VTE adversely influenced several outcomes including mortality. This association was most pronounced in the case of lung resections, which is expected given the pathophysiology of VTE. Alternatively, VTE may develop in the setting of other complications that similarly result in adverse outcomes. Taken together, our results suggest the need for disease-specific recommendations and tracking of compliance with VTE prophylaxis.

With increasing attention to value-based care, several investigators have examined resource use ascribed to VTE following oncologic procedures [6,30]. In a recent report, Mallick and colleagues found increased rates of readmission following diagnosis of VTE after hospital discharge across a wide array of cancer operations [6]. In the present work, we directly examined the impact of VTE during index hospitalization and found significant associations between its development and hospitalization costs, nonhome discharge, and all-cause readmission. Among all operations examined, VTE occurrence increased the overall probability of nonhome discharge by 42.0% and risk of 90-day readmission by 13.1%. Analysis of cumulative risk over time revealed that patients who developed VTE had a nearly 50% chance of readmission in the 90 days following discharge. Moreover, VTE carried a significant financial impact, increasing index hospitalization costs by an estimated $40,000 and readmission-associated costs by $3,200. These observations are congruent with Lyman et al who reported a $20,000 difference in cost associated with VTE patients hospitalized for cancer treatment broadly [30]. Our findings add to the growing body of literature advocating for improved identification of oncologic patients at high risk for unplanned rehospitalization and better access to urgent cancer care to improve long-term patient outcomes [31].

The present study has several important limitations inherent to the nature of the NRD as an administrative database. Clinical data such as VTE prophylaxis measures, tumor grading, laboratory values, and adjuvant therapy regimen were unavailable for analysis. Additionally, we could not evaluate the potential impact of the intraoperative course. We also acknowledge that the NRD captures data from hospitals in 28 states and may underestimate the true burden of readmissions. As the database only accounts for events occurring during inpatient episodes, mortality outside of a hospital setting could not be identified. Despite these limitations, we used validated data practices recommended by HCUP and robust statistical methods to mitigate the risk of bias.

In summary, we found VTE to be independently associated with inferior outcomes following all examined major thoracic and abdominal cancer operations, with increased rates of mortality and hospital length of stay. The effect of VTE extended beyond the perioperative period and was associated with greater risk of nonhome discharge and readmission. VTE was also associated with significant resource utilization, increasing index hospitalization costs by an estimated $40,000 and readmission costs by approximately $3,200. Compared to several patient- and hospital-level factors, type of cancer resection appears to have the greatest influence on the development of VTE. These findings underscore the impact of VTE in oncologic surgery and the importance of continued vigilance and procedure-specific prophylaxis measures.

The following are the supplementary data related to this article.

Supplementary Table 1 Unadjusted index hospitalization outcomes stratified by VTE incidence and resection type Supplementary Table 1

Supplementary Table 2 Unadjusted postdischarge outcomes stratified by VTE incidence and resection type Supplementary Table 2

Supplementary Figure 1 Flow diagram of patient selection Supplementary Figure 1

## Author Contribution

Chelsea Pan: Conceptualization, Formal analysis, Visualization, Writing – original draft.

Yas Sanaiha: Methodology, Formal analysis, Writing – original draft.

Joseph Hadaya: Conceptualization, Writing – review & editing.

Cory Lee: Conceptualization, Writing – review & editing.

Zachary Tran: Conceptualization, Writing – review & editing.

Peyman Benharash: Conceptualization, Methodology, Writing – original draft, Writing – review & editing, Resources, Supervision.

## Conflict of Interest

None.

## Funding Source

None.

## Ethics Approval

This study was deemed exempt from full review by the Institutional Review Board at the University of California, Los Angeles.
