# Supplementary material for: Venous thromboembolism in cancer surgery: A report from the nationwide readmissions database
Source: Surg Open Sci. 2022 May 7;9:58–63. doi: 10.1016/j.sopen.2022.04.005 (PMC9166654; doi:10.1016/j.sopen.2022.04.005)
Supplement: Supplementary Table 1 — Unadjusted index hospitalization outcomes stratified by VTE incidence and resection type [file mmc1.pptx]

## Slide 1
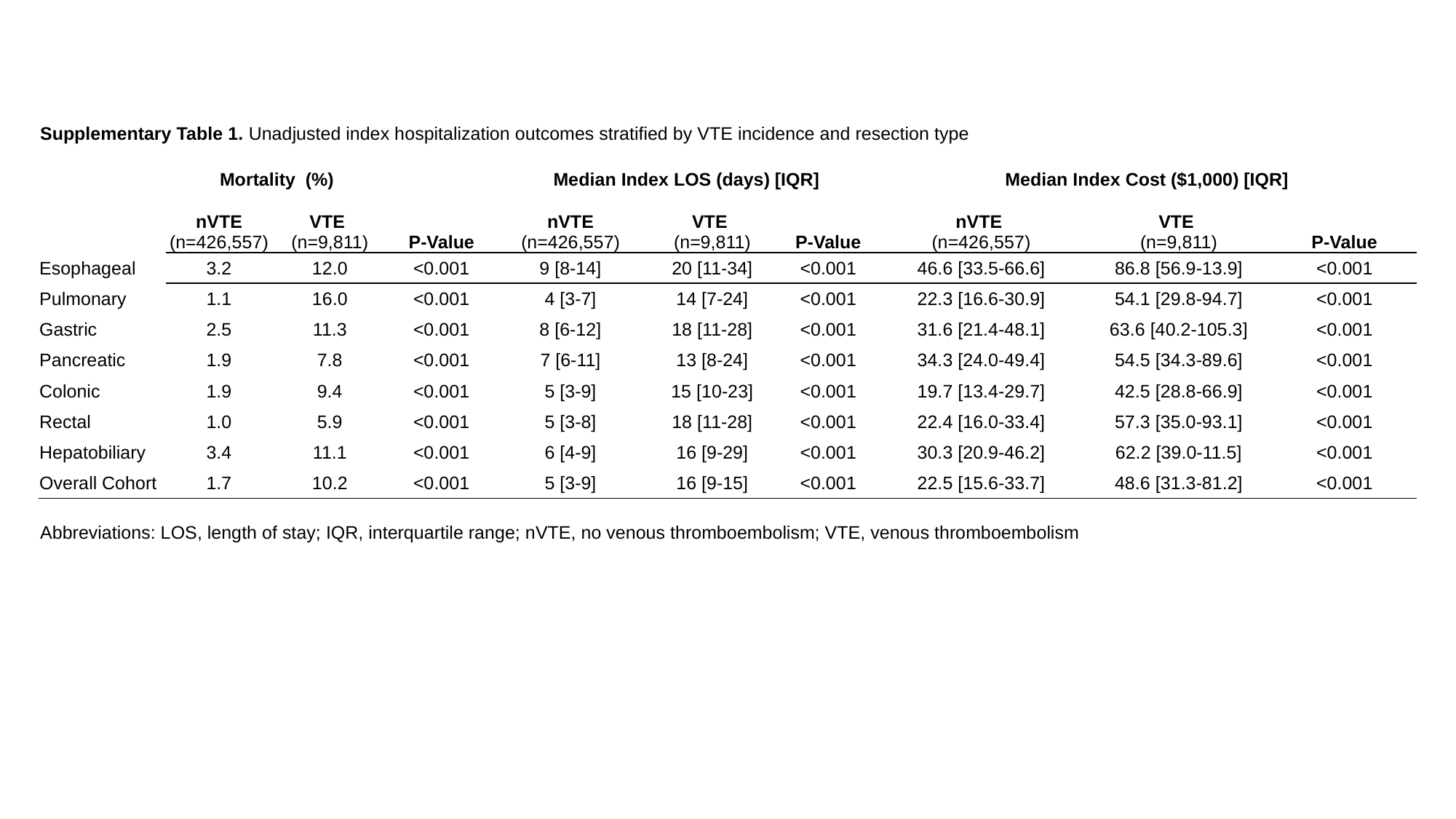

Supplementary Table 1. Unadjusted index hospitalization outcomes stratified by VTE incidence and resection type
| | Mortality (%) | | | Median Index LOS (days) [IQR] | | | Median Index Cost ($1,000) [IQR] | | |
| --- | --- | --- | --- | --- | --- | --- | --- | --- | --- |
| | nVTE (n=426,557) | VTE (n=9,811) | P-Value | nVTE (n=426,557) | VTE (n=9,811) | P-Value | nVTE (n=426,557) | VTE (n=9,811) | P-Value |
| Esophageal | 3.2 | 12.0 | <0.001 | 9 [8-14] | 20 [11-34] | <0.001 | 46.6 [33.5-66.6] | 86.8 [56.9-13.9] | <0.001 |
| Pulmonary | 1.1 | 16.0 | <0.001 | 4 [3-7] | 14 [7-24] | <0.001 | 22.3 [16.6-30.9] | 54.1 [29.8-94.7] | <0.001 |
| Gastric | 2.5 | 11.3 | <0.001 | 8 [6-12] | 18 [11-28] | <0.001 | 31.6 [21.4-48.1] | 63.6 [40.2-105.3] | <0.001 |
| Pancreatic | 1.9 | 7.8 | <0.001 | 7 [6-11] | 13 [8-24] | <0.001 | 34.3 [24.0-49.4] | 54.5 [34.3-89.6] | <0.001 |
| Colonic | 1.9 | 9.4 | <0.001 | 5 [3-9] | 15 [10-23] | <0.001 | 19.7 [13.4-29.7] | 42.5 [28.8-66.9] | <0.001 |
| Rectal | 1.0 | 5.9 | <0.001 | 5 [3-8] | 18 [11-28] | <0.001 | 22.4 [16.0-33.4] | 57.3 [35.0-93.1] | <0.001 |
| Hepatobiliary | 3.4 | 11.1 | <0.001 | 6 [4-9] | 16 [9-29] | <0.001 | 30.3 [20.9-46.2] | 62.2 [39.0-11.5] | <0.001 |
| Overall Cohort | 1.7 | 10.2 | <0.001 | 5 [3-9] | 16 [9-15] | <0.001 | 22.5 [15.6-33.7] | 48.6 [31.3-81.2] | <0.001 |
Abbreviations: LOS, length of stay; IQR, interquartile range; nVTE, no venous thromboembolism; VTE, venous thromboembolism
