# Supplementary material for: Venous thromboembolism in cancer surgery: A report from the nationwide readmissions database
Source: Surg Open Sci. 2022 May 7;9:58–63. doi: 10.1016/j.sopen.2022.04.005 (PMC9166654; doi:10.1016/j.sopen.2022.04.005)
Supplement: Supplementary Table 2 — Unadjusted postdischarge outcomes stratified by VTE incidence and resection type [file mmc2.pptx]

## Slide 1
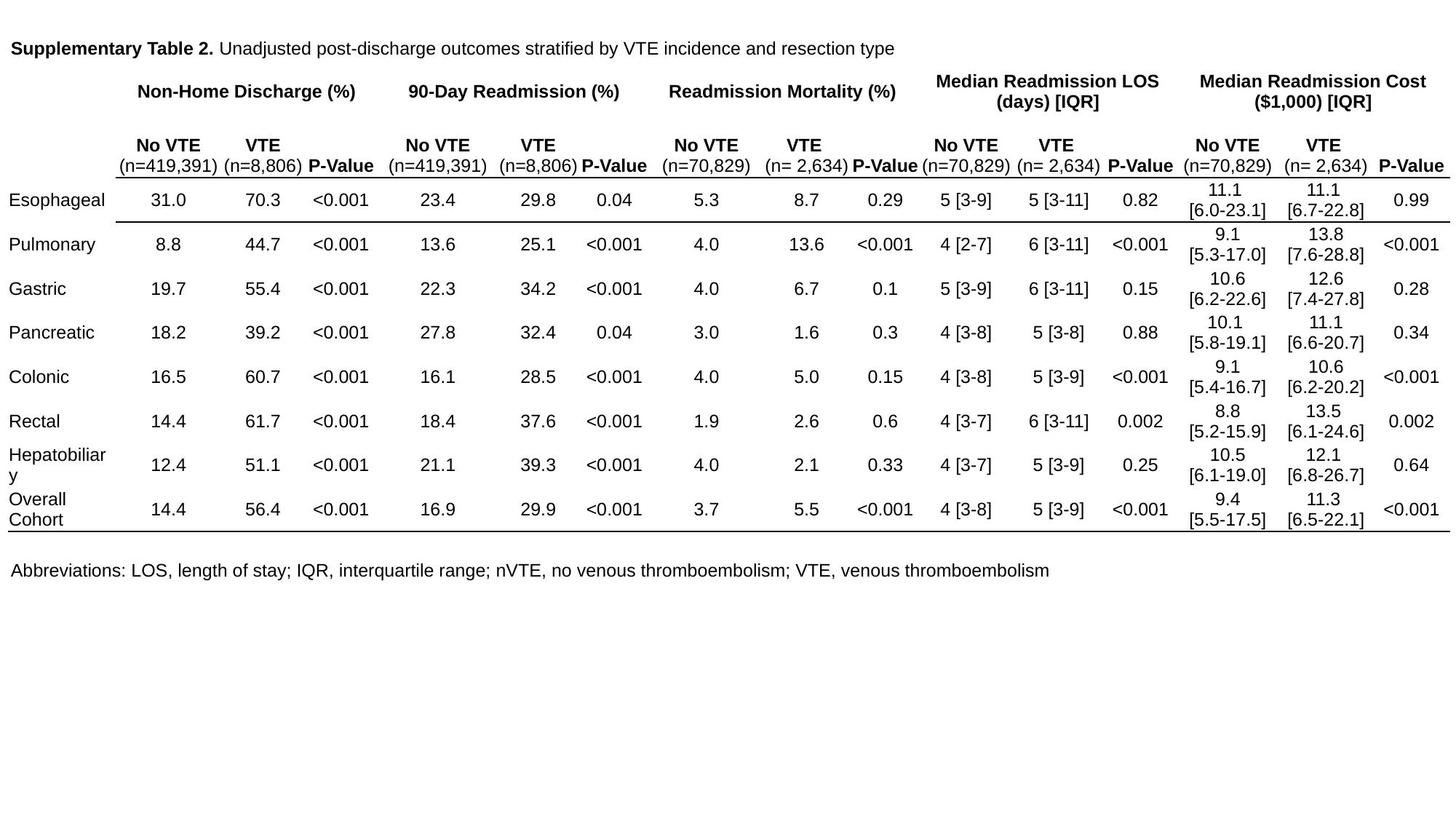

| | Non-Home Discharge (%) | | | 90-Day Readmission (%) | | | Readmission Mortality (%) | | | Median Readmission LOS (days) [IQR] | | | Median Readmission Cost ($1,000) [IQR] | | |
| --- | --- | --- | --- | --- | --- | --- | --- | --- | --- | --- | --- | --- | --- | --- | --- |
| | No VTE (n=419,391) | VTE (n=8,806) | P-Value | No VTE (n=419,391) | VTE (n=8,806) | P-Value | No VTE (n=70,829) | VTE (n= 2,634) | P-Value | No VTE (n=70,829) | VTE (n= 2,634) | P-Value | No VTE (n=70,829) | VTE (n= 2,634) | P-Value |
| Esophageal | 31.0 | 70.3 | <0.001 | 23.4 | 29.8 | 0.04 | 5.3 | 8.7 | 0.29 | 5 [3-9] | 5 [3-11] | 0.82 | 11.1 [6.0-23.1] | 11.1 [6.7-22.8] | 0.99 |
| Pulmonary | 8.8 | 44.7 | <0.001 | 13.6 | 25.1 | <0.001 | 4.0 | 13.6 | <0.001 | 4 [2-7] | 6 [3-11] | <0.001 | 9.1 [5.3-17.0] | 13.8 [7.6-28.8] | <0.001 |
| Gastric | 19.7 | 55.4 | <0.001 | 22.3 | 34.2 | <0.001 | 4.0 | 6.7 | 0.1 | 5 [3-9] | 6 [3-11] | 0.15 | 10.6 [6.2-22.6] | 12.6 [7.4-27.8] | 0.28 |
| Pancreatic | 18.2 | 39.2 | <0.001 | 27.8 | 32.4 | 0.04 | 3.0 | 1.6 | 0.3 | 4 [3-8] | 5 [3-8] | 0.88 | 10.1 [5.8-19.1] | 11.1 [6.6-20.7] | 0.34 |
| Colonic | 16.5 | 60.7 | <0.001 | 16.1 | 28.5 | <0.001 | 4.0 | 5.0 | 0.15 | 4 [3-8] | 5 [3-9] | <0.001 | 9.1 [5.4-16.7] | 10.6 [6.2-20.2] | <0.001 |
| Rectal | 14.4 | 61.7 | <0.001 | 18.4 | 37.6 | <0.001 | 1.9 | 2.6 | 0.6 | 4 [3-7] | 6 [3-11] | 0.002 | 8.8 [5.2-15.9] | 13.5 [6.1-24.6] | 0.002 |
| Hepatobiliary | 12.4 | 51.1 | <0.001 | 21.1 | 39.3 | <0.001 | 4.0 | 2.1 | 0.33 | 4 [3-7] | 5 [3-9] | 0.25 | 10.5 [6.1-19.0] | 12.1 [6.8-26.7] | 0.64 |
| Overall Cohort | 14.4 | 56.4 | <0.001 | 16.9 | 29.9 | <0.001 | 3.7 | 5.5 | <0.001 | 4 [3-8] | 5 [3-9] | <0.001 | 9.4 [5.5-17.5] | 11.3 [6.5-22.1] | <0.001 |
Supplementary Table 2. Unadjusted post-discharge outcomes stratified by VTE incidence and resection type
Abbreviations: LOS, length of stay; IQR, interquartile range; nVTE, no venous thromboembolism; VTE, venous thromboembolism
