# Supplementary material for: Venous thromboembolism in cancer surgery: A report from the nationwide readmissions database
Source: Surg Open Sci. 2022 May 7;9:58–63. doi: 10.1016/j.sopen.2022.04.005 (PMC9166654; doi:10.1016/j.sopen.2022.04.005)
Supplement: Supplementary Figure 1 — Flow diagram of patient selection [file mmc3.docx]

All patients undergoing selected operations in NRD

2,351,892 patients

Removed patients <18 years of age and missing key data (age, sex, index hospitalization mortality and cost)

54,296 patients

2,297,596 patients

Removed patients without corresponding cancer diagnoses

1,687,627 patients

609,969 patients

Removed patients undergoing multiple cancer resections

25,517 patients

584,452 patients

Removed patients with chronic VTE diagnosis

1,403 patients

583,049 patients

Removed patients discharged between October and December of each calendar year

146,681 patients

436,368 patients

- VTE **(nVTE)**

426,557 patients (97.8%)

+ VTE **(nVTE)**

9,811 patients (2.2%)

Supplementary Figure 1. Flow diagram of patient selection
